# An unusual case of abdominal mycobacterial infection: Case report and literature review

**DOI:** 10.4102/sajhivmed.v20i1.993

**Published:** 2019-08-28

**Authors:** Pieter Ekermans, Rene de Gama, Celeste Kock, Ebrahim Hoosien, Tomas Slavik, Terry Marshall, Craig Corcoran, Jakko van Ingen

**Affiliations:** 1 Department of Microbiology, National Reference Laboratory, AMPATH Laboratories, Centurion, South Africa; 2 Department of Paediatrics, Netcare Unitas Hospital, Centurion, South Africa; 3 Department of Paediatrics, Mediclinic Midstream Hospital, Midstream, South Africa; 4 Department of Histology, AMPATH Laboratories, Pretoria, South Africa; 5 Department of Molecular Medicine, National Reference Laboratory, AMPATH Laboratories, Centurion, South Africa; 6 Department of Medical Microbiology, Radboud University Medical Center, Nijmegen, the Netherlands

**Keywords:** Mycobacterium genavense, Non-tuberculous mycobacterium, 16S rRNA sequence analysis, Line-probe assay, Fastidious, Retractile mesenteritis

## Abstract

This article presents a case of an HIV-infected paediatric patient with an unusual *Mycobacterium genavense* infection with predominantly abdominal organ involvement.

The patient is a chronically ill 8-year-old boy from Limpopo province in South Africa, living with his adoptive parents. He was born prematurely at 7 months’ gestation, weighing 1.9 kg. There was a history of recent travel to the Kruger National Park, and to India 6 months prior to admission. All his vaccinations were up to date on history, but this was never confirmed. He was markedly underweight for his age (with weight-for-age and height-for-age *z* scores of −2 and −1, respectively, and body mass index of 13) with a 2-year history of abdominal distension, diarrhoea, failure to thrive and drenching night sweats. There was no history of chronic cough. His treatment up to the time of admission included nutritional and iron supplements and repeated courses of antibiotics. No clinical improvement was achieved with this management.

The patient was referred to a paediatric gastroenterologist in October 2016. He was acutely ill, severely wasted (17 kg) and pyrexial (39 °C). He was clinically pale with a tachycardia and mild oedema of his lower limbs. Hepatosplenomegaly was detected, although there was no peripheral lymphadenopathy. His abdomen was severely distended, and he had recurrent diarrhoea and vomiting with marked intolerance of all foods.

Abdominal computed tomography (CT) scan and ultrasound revealed massively enlarged intra-abdominal lymph nodes (see Figure 1) with a moth-eaten appearance of the spleen. Prominent collateral circulation was seen, which was suggestive of portal hypertension.

**FIGURE 1:**
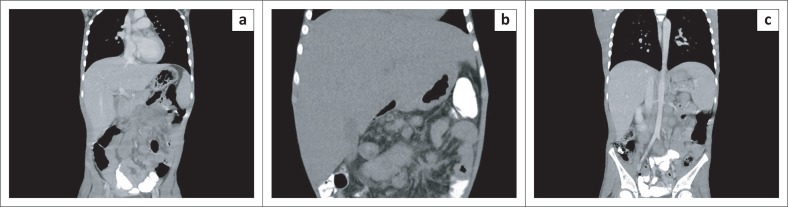
Radiology (computed tomography coronal images of the chest, abdomen and pelvis, a–c). Multiple conglomerate nodal masses are seen along the mesenteric, aorta, iliac and para-aortic nodal chains. There are also enlarged nodes in the porta hepatis. Several of the upper abdominal mesenteric nodes demonstrate low density, compatible with central necrosis. Diffuse hepatomegaly with no discrete lesion is seen.

Laboratory investigations confirmed that the patient was HIV-infected with a CD4 count of 59 cells/μL (7%) and HIV viral load of 453 780 copies/mL (log_10_ 5.66). Further testing revealed mildly elevated liver enzymes. Moderate proteinuria was present and the faecal α-1 antitrypsin result was in keeping with a protein-losing enteropathy. His blood count showed microcytic hypochromic anaemia, with the iron function studies reflecting a pattern of reticuloendothelial iron blockade (see Table 1).

**TABLE 1:** Laboratory results.

Biochemical/haematological parameter	Patient value		Reference range
	October 2016	March 2017	
ALP	91 IU/L	445 IU/L	< 300 IU/L
GGT	58 IU/L	1098 IU/L	< 17 IU/L
ALT	44 IU/L	92 IU/L	< 39 IU/L
AST	84 IU/L	132 IU/L	< 51 IU/L
Albumin	20 g/L	-	38 g/L – 54 g/L
Globulin fraction	57 g/L	-	22 g/L – 36 g/L
Urine protein:creatinine ratio	61 mg/mmol	-	< 20 mg/mmol
Faecal α-1 antitrypsin	1.72 mg/g	-	0.43 mg/g – 1.47 mg/g
Haemoglobin	8.8 g/dL	-	11.5 g/dL – 15.5 g/dL
MCV	75.6 fL	-	77.0 fL – 95.0 fL
MCH	24.4 pg	-	25.0 pg – 33.0 pg
Iron	6.2 mmol/L	-	4.8 mmol/L – 17.2 mmol/L
Transferrin	1.5 g/L	-	1.3 g/L – 3.1 g/L
Percentage saturation	17%	-	17% – 42%
Ferritin	495 ng/mL	-	7 ng/mL – 140 ng/mL

ALP, alkaline phosphatase; GGT, gamma-glutamyl transferase; ALT, alanine aminotransferase; AST, aspartate aminotransferase; MCV, mean corpuscular volume; MCH, mean corpuscular hemoglobin.

The differential diagnosis included tuberculosis or lymphoma. Over a period of 7 months, endoscopically and surgically obtained biopsy material was submitted for histology (see Table 2). Histological images of the duodenum and a lymph node are shown in Figure 2. The findings were consistent with non-tuberculous mycobacterial infection.

**FIGURE 2:**
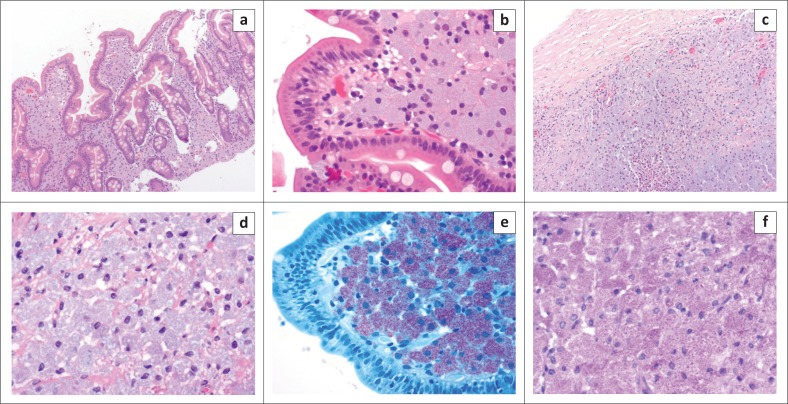
Histological images of the duodenum and lymphnode. (a) Histology of the duodenal mucosa showing lamina propria expansion by histiocyte sheets (hematoxylin and eosin, 100×). (b) Duodenal villus filled with abundant organism-containing histiocytes (hematoxylin and eosin, 400×). (c) Lymph node replaced by sheets of histiocytes with scattered intervening inflammatory cells (hematoxylin and eosin, 100×). (d) Lymph node histiocytes revealing finely granular to foamy cytoplasm, engorged with bacilli (hematoxylin and eosin, 400×). (e) Ziehl–Neelsen stain demonstrating large numbers of acid-fast bacilli in duodenal histiocytes (400×). (f) Periodic acid-Schiff stain showing granular positivity in the histiocyte intracytoplasmic organisms (400×).

**TABLE 2:** Histology results.

Date	Biopsy material	Histological findings
12 October 2016	Mesenteric lymphnode	Lymphadenitis; acid-fast bacilli (AFB) (short morphology); Periodic Acid Schiff (PAS) positive; poorly formed, focal granulomata
	Duodenum and caecum	Granulomatous duodenitis and colitis; histiocytes packed with acid-fast and PAS positive bacilli (small)
08 March 2017	Duodenum	Duodenitis; weak epithelioid granuloma formation; histiocytes noted again as on previous biopsy material
	Liver and intra-abdominal lymphnode	Liver: granulomatous hepatitis (moderate to well-formed epitheloid granulomata; scattered intracytoplasmic AFB in portal granulomata); intra-abdominal lymphnode: lymphadenitis; histiocytes filled with acid-fast and weakly PAS positive bacilli
06 May 2017	Duodenum	The results were similar to previous biopsies, although the infiltrate of AFB containing histiocytes was less impressive in this specimen

Further testing of the biopsy specimens included mycobacterial cultures, all of which showed no growth. Acid-fast bacilli (AFB) were noted during the processing of the tissue samples obtained from the intra-abdominal lymph nodes on 12 October 2016, and again in biopsy material obtained on 08 March 2017. Microscopy performed on a stool sample on 26 March 2017 also showed acid-fast organisms with a coccoid appearance. The polymerase chain reaction (PCR) assay for *Mycobacterium avium* complex was negative (artus® Mycobac. diff. LC PCR from Qiagen, Germany). Numerous PCR assays for *Mycobacterium tuberculosis* complex were negative, which include Nanogen (Nanogen Inc., San Diego, CA, USA), BD MAX (BD Diagnostics, Sparks, MD) and the Xpert® MTB/RIF assay (Cepheid Inc., CA, USA). The urinary lipoarabinomannan test conducted on 22 March 2017 was positive.

A cytomegalovirus (CMV) viraemia of 3974 copies/mL (log_10_ 3.60) was measured in March 2017 together with a colon biopsy that was PCR positive for CMV. The Epstein Barr viral load at the time was 9318 copies/mL (log_10_ 3.97).

Anti-mycobacterial treatment was started (rifampicin, isoniazid, ethambutol, pyrazinamide and clarithromycin) followed 4 weeks later with antiretroviral therapy (abacavir, lamivudine and efavirenz). The patient’s HIV viral load was undetectable at 3 months, and his CD4 count at that stage was 101 cells/μL (6%). Clinically and radiologically, however, there was no improvement in his abdominal signs and symptoms. Malabsorption and refeeding syndrome was considered, and all treatment, including ganciclovir, was given intravenously. His antiretroviral medication was temporarily suspended until oral feeding could be tolerated. Immune reconstitution inflammatory syndrome (IRIS) was considered, and methylprednisone was initiated (1 mg/kg/dose) for 4 weeks, after which the dosage was tapered and stopped. The patient was given a period of bowel rest and free drainage, after which he was placed on an elemental diet. There was no clinical improvement and liver dysfunction worsened (see Table 1), and hence a decision was made to stop rifampicin, isoniazid and pyrazinamide.

In May 2017, a diagnosis of *Mycobacterium genavense* was made, based on sequencing of a mycobacterial 16S rRNA PCR product. This identity was subsequently confirmed using the HAIN Lifescience GenoType (Nehren, Germany) Mycobacterium AS assay that was performed directly on a histology specimen from May 2017. As a result of this finding, and in consultation with an infectious disease specialist and microbiologist, treatment was changed to include moxifloxacin, azithromycin and rifabutin for 2 years, with amikacin for the first 3 months. Methylprednisone was also restarted. Antiretroviral therapy, together with cotrimoxazole prophylaxis, was continued.

The patient’s response to the new regimen was slow, and initially he was unable to tolerate food. Insertion of a nasogastric tube was required for continuous feeds together with total parenteral nutrition. At the time of writing this article (17 months of treatment completed), his clinical response had improved. He was able to tolerate small regular meals with no nausea, vomiting or diarrhoea. His weight gain had been slow (now up to 20 kg) despite nutritional supplementation. The hepatosplenomegaly and abdominal distension had improved markedly, and his HIV remains virologically suppressed.

## Ethical consideration

Dr R. de Gama obtained consent from the patient’s parents to publish this case report.

## Discussion

The first case of *M. genavense* was described in 1987 in the clinical setting of acquired immunodeficiency syndrome (AIDS);^1^ the bacterium is closely related to *Mycobacterium simiae*.^2^ *Mycobacterium genavense* has been recovered from dogs, cats, rabbits, monkeys, ferrets and a variety of birds (parrots, budgerigars, amazons, flycatchers, zebra finches, hoopers, parakeets, parrotlets and waxwings).^1,3^ It is the most common cause of psittacine mycobacteriosis.^4^ It has also been isolated from the respiratory and gastrointestinal tracts of healthy individuals^5,6^ and from tap water.^7^ This organism has not been recovered from soil,^8^ and no human-to-human transmission has been described.^9^

Human isolates have been recovered from cultures of blood, bone marrow, liver, spleen and other tissues.^8^ Faeces may also show a large amount of AFB.^4,9^

*Mycobacterium genavense* is a fastidious non-tuberculous mycobacterium requiring special supplementation with mycobactin J, adjusted pH and incubation temperature of 37 °C – 45 °C for isolation from culture specimens.^2,10^ Use of Middlebrook 7H11 solid medium supplemented with sheep blood and charcoal acidified to pH 6.2+/−0.2 has also been noted.^11^ Thompson and colleagues^12^ used the BACTEC pyrazinamide test medium and determined pH 5.5 to yield the best growth for susceptibility testing. The duration of incubation is typically 8–12 weeks.^8^ About 30% – 50% of cases are identified after prolonged incubation.^10^

### Clinical presentation

Most clinical *M. genavense* isolates have been cultured from patients with advanced HIV infection, especially in the pre-highly active antiretroviral therapy (HAART) era.^9,10^ *Mycobacterium genavense* presents similarly to *M. avium* complex: It is encountered in patients with CD4 counts < 100 cells/mm^3^; has a high affinity for the abdomen; and causes abdominal pain, diarrhoea, hepatosplenomegaly, lymph node enlargement and sometimes ascites.^6^ *Mycobacterium genavense* should be considered in HIV-infected patients with suspected disseminated *M. avium* complex, but whose routine cultures are negative.^8^ Immune reconstitution inflammatory syndrome may occur in patients on HAART infected with this pathogen, and symptoms may paradoxically worsen, leading to severe complications.^6^ Disseminated *M. genavense* infection account for 4% – 13% of non-tuberculous mycobacteria in HIV-infected patients.^13^ Descriptions of documented clinical cases with *M. genavense* in patients with HIV and/or AIDS have been similar to our patient in presentation and course of disease.

Recently, cases of *M. genavense* have been described in patients with non-HIV-related immunological pathology.^10^ Risk categories in which clinical disease due to *M. genavense* occurs include patients with HIV and/or AIDS,^4,5,6,13,14^ lymphoproliferative disorders,^15,16^ solid organ and allogeneic stem cell transplant patients,^10,17,18,19^ patients receiving chronic steroids in combination with other immunomodulating drugs^20^ and patients with primary immunodeficiency diseases.^21,22,23^

Other diseases that may be associated with *M. genavense* infection include sarcoidosis, hyper-IgE syndrome and auto-immune disorders (systemic lupus erythematosus and myasthenia gravis).^9,24,25,26,27^

While most patients with *M. genavense* infection have immunological pathology, a case of disseminated *M. genavense* infection in a healthy Japanese boy has also been described. Computed tomography of the abdomen showed intestinal wall thickening from the ileocecum to the ascending colon, as well as small intestinal dilation and ascites. The only possible risk factor appeared to be exposure to pets, including dogs, rabbits, turtles and tropical fish.^28^

The clinical presentation of cases with *M. genavense* appears mostly to be disseminated involving abdominal organs. Less common presentations include pleuropulmonary, cutaneous, central nervous system and genital tract involvement.^9,29^ Pulmonary involvement may include cavitations and reticular–nodular infiltrates on chest X-ray. A few cases of pulmonary *M. genavense* disease have been documented.^20^

The laboratory diagnosis of *M. genavense* infections usually relies on detection by molecular methods. Mycobacterial 16S rRNA sequence analysis is often used for the confirmation of the diagnosis.^9,10^ The GenoType Mycobacterium AS line probe assay (Hain Lifescience, Nehren, Germany) can also be used, but it cannot distinguish between *M. genavense* and *Mycobacterium triplex*. It is validated for use on cultured material. We ran this line-probe assay method directly on the duodenal biopsy according to the standard GenoType MTBDRplus protocol and were able to generate an interpretable banding pattern in our patient.

### Treatment

*In vitro* susceptibility data are limited because of the extreme fastidiousness of the organism, requiring special supplementation, an acid pH and prolonged incubation.^8,9^ Available data suggest that most isolates are susceptible to macrolides, rifamycins, fluoroquinolones and aminoglycosides (amikacin and streptomycin).^8^ *Mycobacterium genavense* is resistant to isoniazid.^9^ Optimal therapy is not determined.^8^ In animal models, a reduction in AFB burden is seen after 15–30 days with clarithromycin and rifampicin and after 30 days with amikacin and ethambutol.^10^ A three- or four-drug regimen is typically suggested. In one case series, a regimen including a macrolide, ethambutol and often rifampicin recorded a favourable outcome in 75% of cases (9/12).^20^ In another case series, the survival rate at 1 year was 72%. In this case series, a treatment regime typically included clarithromycin, ethambutol and rifabutin, and sometimes also a fluoroquinolone or amikacin.^27^

Multidrug therapies that include clarithromycin appear to be more effective than those without clarithromycin.^8^ Ethambutol, despite limited *in vitro* activity against *M. genavense*,^8,12^ is included in treatment regimens of many documented cases of *M. genavense* infections.^20,27^ In our patient, ethambutol was given for a total of 10 months as part of the initial treatment. Older literature refers to the use of clofazimine.^30,31,32^ This drug was used less frequently for the treatment of disseminated non-tuberculous mycobacterial disease after a clinical study found that clofazimine in combination with clarithromycin and ethambutol was associated with increased mortality in disseminated *M. avium* complex infections in patients with AIDS.^33^ The use of prednisone has been advocated to reduce local inflammation and compressive effects of the affected organs. In one study, it was used for 10 months.^6^ A case series where steroid therapy was included did not describe worse outcomes.^27^

#### Treatment duration and follow-up

The documented cases of *M. genavense* disease indicate that the duration of treatment should be prolonged to more than 12–27 months.^10,21^ To make recommendations on the termination of treatment for these cases is therefore challenging. Due to the fastidious nature of the mycobacterium, treating cases until 12 months culture negativity^8^ is problematic and treating for as long as the immunodeficiency is present has resulted in life-long treatment in some patients.^20^ Mycobacterial blood culture (with prolonged incubation) and stool AFB where appropriate may be of value as markers of treatment response.

Follow-up radiological investigation, especially if CT is used, increases the risks associated with radiation exposure. Some authors suggest an X-ray and high-resolution CT at baseline prior to the commencement of therapy.^34^ Follow-up radiology should be considered together with clinical assessment.^34^ Follow-up with repeat biopsies from the affected organs to compare with initial histology reports might be another option^6^; although as granulomas persist much longer than the infection/disease, this might be a poor marker of response. Rebiopsy for histology and culture may be considered when treatment failure is suspected.

### Complications

A poorly understood pathogen-specific syndrome similar to retractile mesenteritis has been described in patients infected with *M. genavense* where chronic fibrosing inflammation is found in the small bowel mesentery. Rarely, chylous ascites may develop.^4,6^ Persistent relapsing infection may occur in patients with profound immunosuppression and high HIV viral loads at initial diagnosis with a large inoculum of *M. genavense* organisms.^6^ A case of an HIV-infected paediatric patient with intestinal lymphangiectasia and protein-losing enteropathy has been described. This patient presented with severe hypogammaglobulinaemia and moderate hypoalbuminaemia. Lymphatic vessel dilatation, small intestinal wall thickening, ascites as well as retroperitoneal and mesenteric adenopathy were seen on abdominal magnetic resonance imaging. Elevated α-1 antitrypsin in stool confirmed the diagnosis of protein-losing enteropathy.^13^ A similar scenario was reported by Tassone and colleagues.^21^ Hyperammonemia was described in a renal transplant case with disseminated *M. genavense* infection.^35^

### Conclusion

The patient described in this case report is illustrative of the difficulties encountered in accurately diagnosing and managing disease caused by non-tuberculous mycobacteria, in this case *M. genavense.* Clinicians and laboratory professionals need to be aware of non-tuberculous mycobacterial infections, particularly encountered in immunocompromised patients and use available molecular diagnostic tools to obtain a diagnosis.

## Teaching points

- *Mycobacterium genavense* should be considered in HIV-infected patients with suspected disseminated *M. avium* complex, but whose routine cultures are negative.
- This organism is the most common cause of psittacine mycobacteriosis.
- The clinical presentation of patients with *M. genavense* usually includes dissemination involving abdominal organs.
- Available molecular diagnostic tools are used to obtain a diagnosis.
- A three- or four-drug regimen is typically suggested for treatment.
- Treatment is prolonged to more than 12–27 months.
